# 金属有机骨架基复合材料在色谱固定相中的应用进展

**DOI:** 10.3724/SP.J.1123.2026.01030

**Published:** 2026-11-08

**Authors:** Yan MA, Jiaping CUI, Lan XIA, Zhining XIA, Qifeng FU

**Affiliations:** 1. 西南医科大学药学院，四川 泸州 646000; 1. School of Pharmacy，Southwest Medical University，Luzhou 646000，China; 2. 重庆大学药学院，重庆 401331; 2. School of Pharmaceutical Sciences，Chongqing University，Chongqing 401331，China; 3. 海南大学海洋科学学院，南海海洋资源利用国家重点实验室，海南 海口 570228; 3. State Key Laboratory of Marine Resource Utilization in South China Sea，School of Marine Sciences，Hainan University，Haikou 570228，China

**Keywords:** 金属有机骨架, 复合材料, 色谱固定相, 分离机制, 综述, metal-organic frameworks, composites, chromatographic stationary phase, separation mechanism, review

## Abstract

金属有机骨架（MOFs）凭借其超高的比表面积、高度有序的可调孔径及丰富的表面化学性质，作为新型色谱固定相或前处理介质展现出广阔的应用前景。然而，纯相MOFs材料在实际色谱应用中常受限于热力学吸附与动力学传质之间的本征矛盾，表现为机械稳定性不足、晶体形貌不规整以及单一微孔结构导致的传质阻力大等问题，难以直接满足复杂基质高效分析的需求。为克服上述局限，构建MOFs基复合材料已成为当前分离科学领域的前沿策略。该策略旨在通过功能组分的结构整合实现优势互补，在利用复合基质增强材料机械强度与分散性的同时，优化传质路径并引入多重相互作用位点以提升分离效能。本文从色谱分离的热力学识别与动力学传质原理出发，系统梳理了核壳型、主客体封装型以及骨架杂化型等复合材料的构筑设计策略，深入剖析了异质界面的协同效应机理。在应用层面，本文重点探讨了此类复合材料在高效液相色谱（HPLC）、毛细管电色谱（CEC）及气相色谱（GC）中的最新研究进展，阐述了复合材料在改善色谱峰形与柱效、增强异构体与手性分子选择性识别，以及提升严苛条件下化学与热稳定性方面的作用规律。最后，针对精准合成、重复性提升及复杂体系定制化应用等挑战进行了展望，以期为理性设计下一代高性能色谱分离介质提供理论参考。

## Abstract (en)

Metal-organic frameworks （MOFs） have attracted substantial attention as prospective media for next-generation chromatographic stationary phases and sample pretreatment matrices. Their utility is primarily attributed to their exceptional specific surface area，highly ordered and tunable pore architectures，and versatile surface chemistry，which theoretically enable precise molecular sieving and specific recognition. Nevertheless，the practical implementation of pristine MOFs is frequently constrained by intrinsic physicochemical defects，particularly the profound conflict between thermodynamic affinity and kinetic mass transfer. From a kinetic perspective，the exclusive microporous nature of most MOFs imposes excessive mass transfer resistance，especially for bulky analytes，leading to peak broadening and low column efficiency as described by the resistance to mass transfer term in the van Deemter equation. Such kinetic limitations are particularly detrimental when analyzing macromolecules or performing high-speed separations，thereby hindering the throughput required for modern analytical workflows. Furthermore，practical deployment is often plagued by inadequate mechanical stability under high-pressure conditions，irregular crystal morphologies that induce significant eddy diffusion，and poor chemical stability in acidic or basic eluents，which significantly limits their direct utility in the high-efficiency analysis of complex matrices. To overcome these obstacles，the fabrication of MOF-based composites has evolved into a pivotal strategy within separation science. By integrating functional components through precise structural engineering，this approach exploits the synergistic complementarity of distinct materials to mask defects while amplifying advantages. This review systematically classifies current construction strategies into three primary architectures including core-shell structures，host-guest encapsulation，and skeleton hybridization. In core-shell architectures，MOF crystals are epitaxially grown on or chemically bonded to rigid microspheres such as silica or magnetic particles. This configuration effectively decouples the mechanical stability provided by the core from the separation performance governed by the shell. This design not only enhances mechanical robustness to withstand high backpressures but also shortens the diffusion path length to significantly improve mass transfer kinetics. Host-guest encapsulation strategies leverage the confinement effect of MOF cavities to act as nanocontainers for enzymes，chiral selectors，or metal nanoparticles. This approach effectively prevents the leaching and aggregation of active guests，thereby preserving their catalytic or recognition activities in complex matrices. Skeleton hybridization strategies，such as the construction of MOF-on-MOF or MOF-COF heterostructures，focus on lattice matching to ensure continuous pore channels across interfaces. This creates complex microenvironments that enhance selectivity through distinct steric and electronic interactions at the interface. In terms of applications，this review critically assesses the performance of these composites across various chromatographic modes. In HPLC，composites featuring hierarchical pore structures are shown to significantly ameliorate peak shapes and separation factors for challenging isomers and chiral drugs. For capillary electrochromatography （CEC），the review highlights how composites allow for the precise regulation of electroosmotic flow （EOF） through surface charge modulation，facilitating the separation of complex biological samples. Regarding gas chromatography （GC），where thermal stability is paramount，composite strategies involving covalent bonding or protective shelling are demonstrated to bolster structural integrity and effectively suppress column bleeding under rigorous thermal gradients. This enhancement is vital for maintaining baseline stability and detector sensitivity during high-temperature programming. Additionally，the role of these composites in sample pretreatment is also discussed，emphasizing their high enrichment factors and anti-interference capabilities in extracting trace targets from complex biological or environmental matrices. Finally，the review envisions future directions for this field. While current research has validated the concept of MOF-based composites，persistent challenges remain regarding the precision synthesis of defect-free interfaces，the enhancement of batch-to-batch reproducibility for commercial-grade production，and the development of smart stimuli-responsive separation media. Moreover，interdisciplinary collaboration between crystal engineering and chromatographic theory is essential to tailor these materials for specific，real-world separation challenges. Addressing these issues will be instrumental in realizing the full potential of MOF-based composites as high-performance separation media.

色谱技术作为现代分析科学的基石，其核心竞争力的提升高度依赖于固定相材料的创新。在分离科学领域，开发兼具高选择性识别能力与优异动力学传质性能的固定相始终是研究者追求的目标。金属有机骨架（MOFs）作为一类由金属节点与有机配体组装而成的晶态多孔材料^［1］^，凭借其超高的比表面积、可调的规整孔道以及丰富的表面化学性质，为实现分子尺度的高效筛分与识别提供了理想的结构基础^［2，3］^。虽然传统的烷基链或极性官能团修饰能够在一定程度上调节分离选择性，但这类修饰往往局限于疏水性或尺寸效应等单一分离机制，难以应对结构日益复杂的分析对象^［4-6］^。

当直接将纯相MOFs用作色谱固定相时，其本征的物理化学缺陷日益凸显。一方面，热力学吸附优势与动力学传质劣势之间的矛盾较为突出^［7，8］^。尽管MOFs的微孔结构提供了良好的分子筛分与特异性吸附能力，但其内部狭窄曲折的孔道极大地限制了溶质分子，特别是大分子分析物的扩散速率^［9］^。根据范德姆特方程，显著的传质阻力会导致色谱峰严重展宽，从而大幅降低柱效^［10］^。另一方面，材料的机械稳定性与流体动力学的适配性不强。大多数常规合成的MOFs形貌不规则且粒径分布较宽，直接填充不仅会引起严重的涡流扩散导致柱效降低，还会造成色谱柱背压过高^［11，12］^。更为关键的是，纯MOFs骨架在高效液相色谱（HPLC）高达数十兆帕的流体剪切力或酸碱、水相体系的侵蚀下，极易发生骨架坍塌或配体流失，严重制约了分离过程的重复性和稳定性。

为了克服上述单一材料的性能瓶颈，构建MOFs基复合材料已成为分离科学领域的重要策略。该类复合策略的核心在于利用不同组分间的优势互补实现材料性能的显著提升。通过将MOFs与高机械强度的无机载体、具备快速传质通道的聚合物骨架^［13］^或具有电子互补效应的框架材料进行异质组装，复合材料有助于实现固定相多重功能的协同优化^［14，15］^。在该体系中，MOF组分主要提供选择性识别位点，而复合基质则承担机械支撑与优化传质路径的任务^［16］^。这种互补机制不仅通过构建有序的扩散通道或缩短传质路径有效降低了传质阻力并改善峰形，更通过多重相互作用位点的集成显著提升了对复杂异构体及痕量目标物的分离度^［17，18］^。

尽管MOFs在色谱领域的应用已有相关论述，但目前鲜有综述从构筑逻辑与界面作用的深度系统探讨MOFs基复合材料在解决色谱关键问题中的独特价值。基于此，本文结合本课题组近年来在该领域的探索与积累，旨在综述MOFs基复合材料在色谱分离领域的最新研究进展。首先将复合材料的构筑策略划分为核壳型、主客体封装型以及骨架杂化型三大类，深入剖析不同构筑策略下的界面作用机理。随后详细阐述这些材料在气相色谱（GC）、HPLC及毛细管电色谱（CEC）中的应用演进，重点揭示复合材料如何通过强化传质过程和提高分离选择性来解决实际分离的难题。最后对该领域在精准合成、机理解释及实用性考察等方面面临的挑战与未来方向进行展望，以期为理性设计下一代高性能色谱分离介质提供理论参考（图1）。

**图1:**
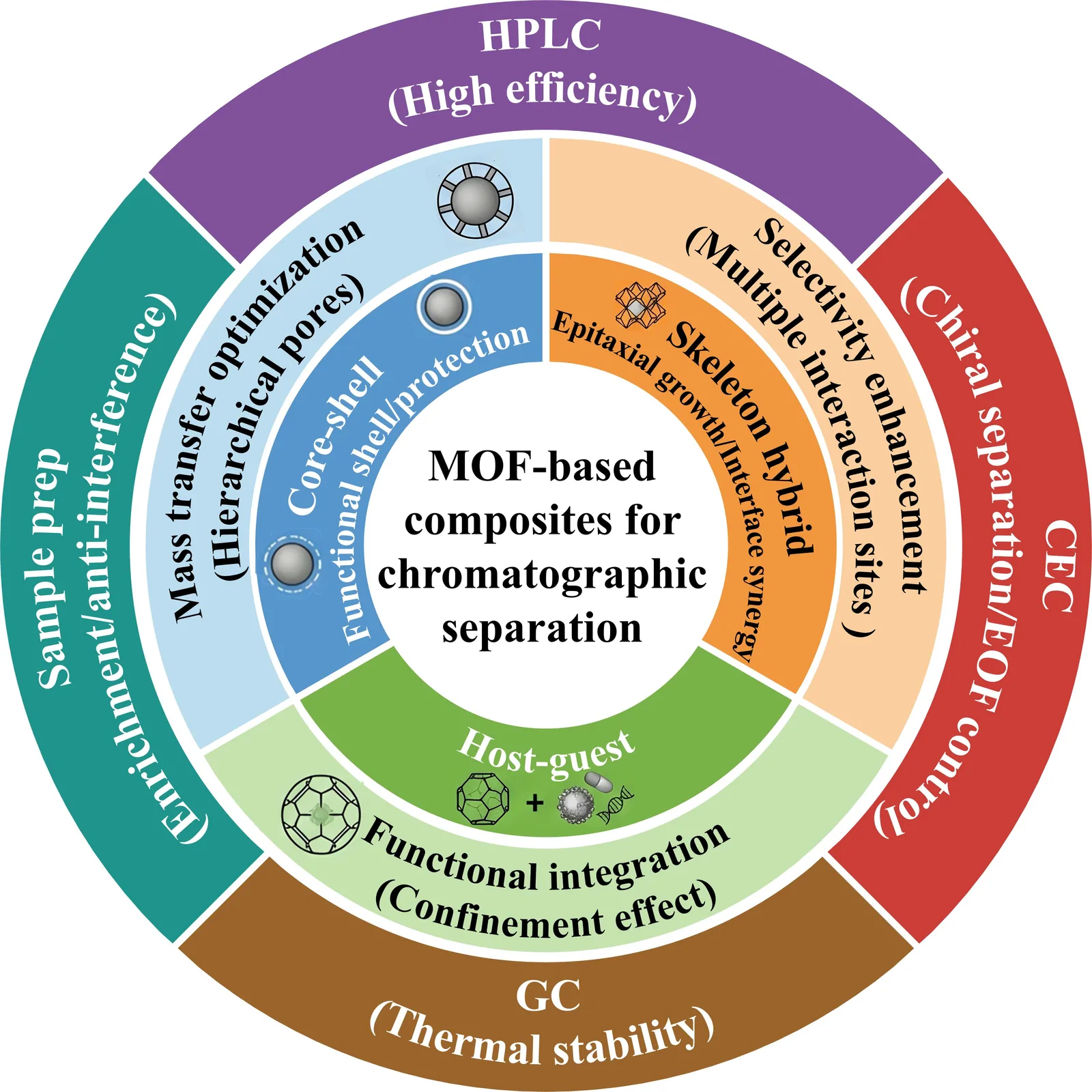
MOFs基复合材料的结构设计及其在色谱中的应用

## 1 MOFs基复合材料的构筑理念与分类原则

针对纯相MOFs材料在实际分离过程中暴露出的机械稳定性差、功能单一及传质受限等本征缺陷^［19］^，复合策略的设计核心在于实现从简单的功能叠加向性能定制的转变。这种演变不仅要求在空间布局上实现MOF组分与载体材料的高度集成，更要求在微观尺度上调控异质界面的相互作用^［20，21］^。从色谱动力学角度分析，引入第二组分能够针对性地优化范德姆特方程中的关键参数。一方面，复合材料通过在MOFs骨架中构建连通的多级孔道结构或引入刚性客体选择剂，可显著缩短溶质分子在固定相内部的扩散路径，从而有效降低传质阻力项（C项）；另一方面，复合组分的引入往往可显著改善材料的晶体形貌规整度与分散性，有助于形成均匀致密的色谱涂层，通过减小涡流扩散项（A项）显著降低理论塔板高度，最终实现色谱峰形的锐化与柱效的提升^［22，23］^。从热力学层面看，异质界面的引入往往会诱导电子云重叠或局部极化效应，从而将分离模式从单一的尺寸筛分扩展为由电荷转移^［24，25］^、疏水相互作用及空间位阻共同驱动的多重模式^［26，27］^。依据复合材料的微观结构特征与组分结合方式，目前的构筑策略主要可分为核壳型、主客体封装型以及骨架杂化型三大类。

### 1.1 核壳型复合材料

核壳构筑策略的核心在于利用异质壳层对核心MOFs进行功能化修饰或物理保护。在该结构体系中，核心MOFs通常作为高比表面积的支撑基底，而外层壳层则负责提供特定的分子识别位点、增强机械化学稳定性或引入多级孔道以优化传质。根据壳层性质的不同，该策略可细分为生物/聚合物软壳修饰与刚性骨架包覆两条路径。

在软壳修饰方面，研究者常利用MOFs表面的活性位点与酶、聚合物等大分子^［28，29］^进行组装，构建兼具生物活性与多孔特性的复合界面。这一策略不仅局限于酶的固定化，目前已进一步拓展至多肽、核酸及蛋白质复合物等更广泛的生物大分子体系，以赋予材料靶向识别或环境响应等新特性。例如，Wang等^［30］^提出了一种原位一锅法策略，在锰钴普鲁士蓝类似物这类MnCo-MOF骨架中杂化聚多巴胺（PDA），并通过在表面进一步修饰巯基化的环状精氨酸-甘氨酸-天冬氨酸（RGD）多肽，成功构建了核壳型纳米凝胶。该复合壳层不仅利用PDA增强了光热转换效率，更通过RGD肽链引入了对肿瘤细胞的主动靶向能力，显著提升了治疗效果。此外，针对核酸类大分子的递送，Alsaiari等^［31］^利用ZIF-8外壳对*CRISPR/Cas9*基因编辑系统（含单导RNA核酸）进行包覆修饰，构建了具有生物相容性的纳米载体。该研究充分利用了ZIF-8壳层中咪唑基团的质子化作用，有效促进了复合物的内体逃逸，解决了生物大分子在胞内递送中的关键动力学屏障。本课题组在此方向也进行了系统探索，致力于解决酶在毛细管微反应器中的固定化难题。早期研究中，本课题组通过原位生长法将微孔UiO-66-NH_2_^［32］^固定于毛细管内壁作为核心载体，随后利用静电相互作用在MOF表面吸附固定乙酰胆碱酯酶层，成功构建了在线酶微反应器并应用于农药残留检测。随着对传质机理认识的深入，针对微孔骨架限制生物大分子负载与底物扩散的瓶颈，本课题组^［33］^进一步开发了具有多级孔结构的HP-UiO-66-NH_2_复合涂层（图2）。在该构筑策略中，通过引入硅烷偶联剂对多级孔MOF（HP-MOF）表面进行共价修饰以增强界面结合力，随后利用介孔尺寸与酶分子的空间匹配性，将黄嘌呤氧化酶牢固限制于骨架内部，形成稳定的生物复合壳层。这种设计在显著提升酶负载量的同时，利用多级孔道有效降低了传质阻力，克服了生物大分子在微孔限域空间内孔道可及性差与传质动力学迟滞的瓶颈。

**图2:**
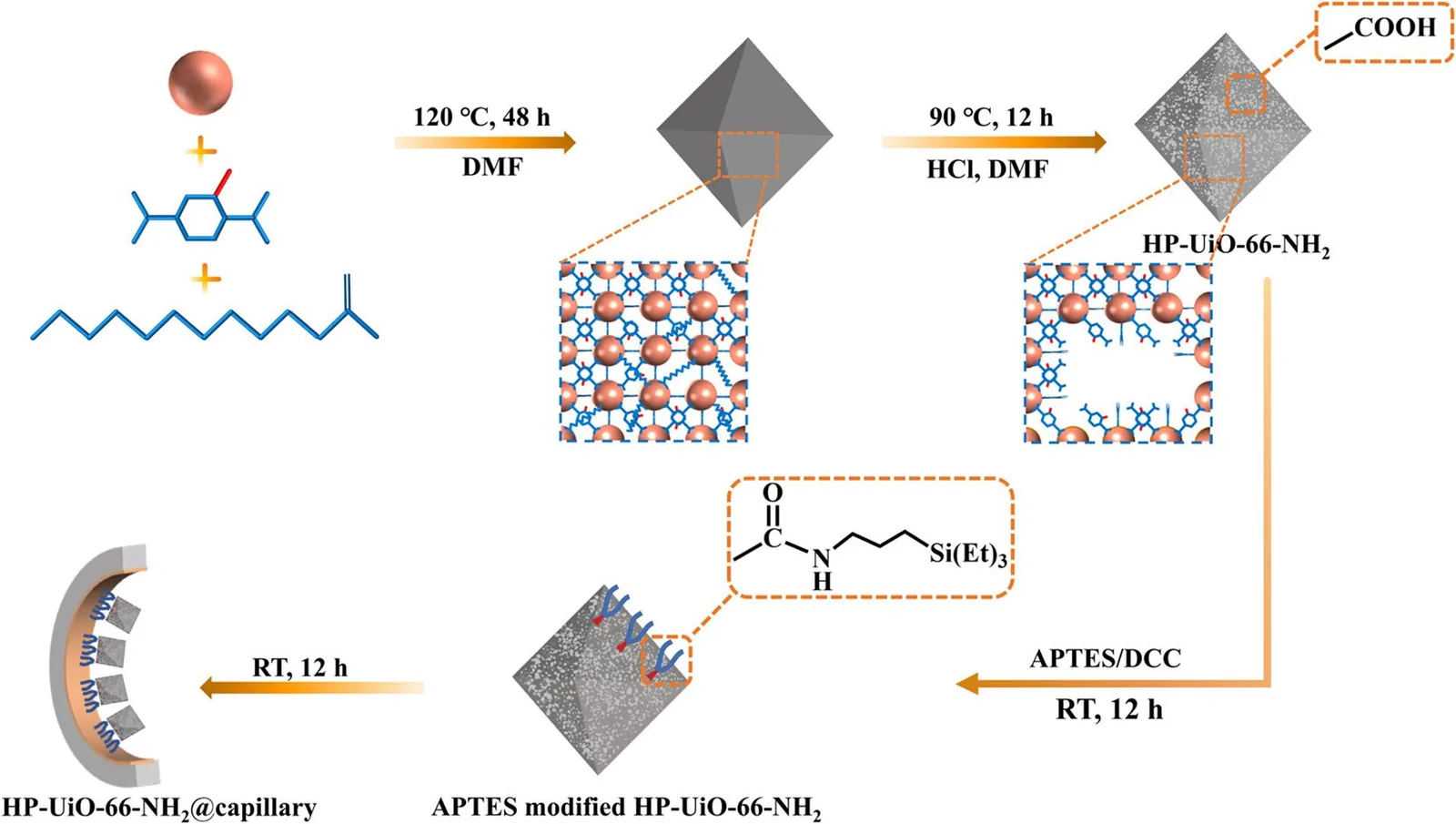
HP-UiO-66-NH_2_涂层毛细管制备示意图^［33］^

在刚性壳层包覆方面，针对MOFs水稳定性差或机械强度不足的问题，在其表面生长疏水性或刚性多孔壳层是更为直接的解决方案。Cai等^［34］^针对具有氨基官能团的MOFs，通过共价锚定方式在其表面原位生长疏水性共价有机骨架（COF）外壳，这种致密的NTU-COF核壳结构显著提高了材料的水接触角，使其在潮湿条件下仍能保持优异的骨架完整性与分离性能。无机硅球也常被用作增强机械强度的核心或壳层。Wan等^［35］^采用先修饰后包覆的策略，利用溶剂热反应诱导UiO-66-NH_2_在氨基化二氧化硅（SiO_2_）微球表面生长。与纯UiO-66-NH_2_颗粒在装填过程中因机械强度不足而易发生破碎，且不规则形貌导致柱背压过高相比^［36］^，这种以SiO_2_为硬核、MOF为活性壳层的复合材料，有效解决了纯MOFs颗粒在高压环境下易破碎的问题。Guo等^［37］^则进一步拓展了这一思路，构建了MOF-235@PEG@Silica复合固定相，该材料利用聚乙二醇与硅球构筑的双重保护壳层，在赋予材料优异机械强度的同时引入了丰富的亲水作用位点，对生物碱的高效分离展现出优异的色谱性能。

### 1.2 主客体封装型复合材料

MOFs基主客体复合材料通过利用MOFs规则的孔道或笼状空腔作为纳米容器，将功能性客体分子封装于其内部^［38，39］^。该策略旨在借助MOFs的限域效应调控客体分子的分散性、防止其团聚或流失，并实现主客体功能的集成。对于小分子客体，常采用物理浸渍与扩散法。Liang等^［40］^通过液相浸渍法将葫芦脲分子封装于MIL-101（Cr）的介孔笼中，成功制备了主客体复合材料并提升了其气体吸附选择性。此外，Chen等^［41］^利用ZIF-8的多孔结构作为纳米反应器，将硅源前驱体引入其孔道，随后通过气相沉积或热处理原位转化为硅纳米点，这种限域生长策略有效避免了活性组分的流失。需要注意的是，虽然封装能保护客体，但需严格控制封装密度以防止过度占据孔道导致传质受阻。对于金属颗粒或生物大分子，则常采用原位合成或原位矿化法。Aijaz等^［42］^利用亲疏水双溶剂策略将金属前驱体溶液限域引入MOFs孔道并原位还原，可制备出分布均匀的金属复合材料。针对荧光纳米粒子易发生猝灭与团聚的问题，本课题组^［43］^采用一步溶剂热法将甲酰胺基碳点原位封装于Eu-MOFs骨架中，利用MOF的刚性笼状结构有效隔离碳点以避免聚集诱导猝灭效应，同时借助骨架的富集作用显著提升了识别灵敏度。此外，为了进一步解决微孔骨架对大尺寸客体的传质限制，本课题组^［44］^还提出了一种“共价键合-瓶中造船”策略，将碲化镉量子点（CdTe QDs）锚定于HP-UiO-66-NH_2_介孔腔内，这种多级孔道设计在大幅提升客体负载量的同时，通过共价连接增强了主客体间的界面稳定性。Li等^［45］^则采用金属阳离子导向的原位组装策略，将金属酞菁封装于bio-MOF-1的孔道内制备了复合固定相。针对生物大分子，Zhou等^［46］^通过孔隙包埋法将牛血红蛋白封装于具有超大介孔的PCN-333（Al）中（图3）。传统的物理吸附法往往导致蛋白分子吸附不牢，在流动相冲刷下极易发生脱附流失，且暴露在外的蛋白易受环境影响而失活^［47，48］^。相比之下，得益于骨架的刚性保护，该主客体复合材料在连续进样分析中表现出优异的长期稳定性，在保持生物亲和性的同时实现了对黄酮类和磺胺类化合物的高效分离。

**图3:**
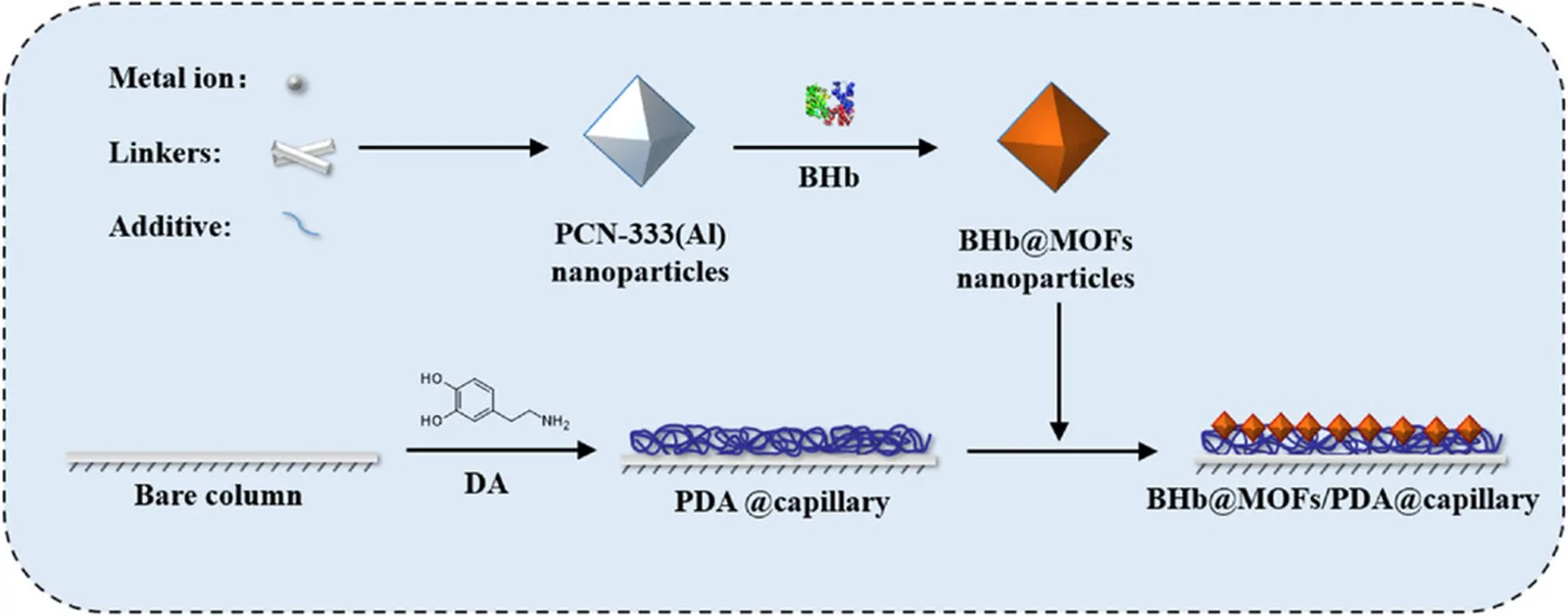
BHb@MOFs/PDA开管毛细管柱的制备过程^［46］^

### 1.3 骨架杂化型复合材料

骨架杂化型复合材料的设计核心在于基于晶格匹配或界面配位原理，在MOFs晶种表面精准构筑具有连续接触界面的异质结构。与简单的物理混合不同，这种基于晶格匹配的外延生长策略有助于维持异质骨架间孔道的贯通性及缓解界面缺陷导致的扩散势垒，从而优化溶质分子跨界面传输的传质动力学行为。更为关键的是，异质组分在界面处的电子云重叠与电荷重分布效应构建了独特的局部极性微环境。这种界面诱导的电子效应旨在调节待测物与固定相之间的范德华力或静电相互作用，使得具有微小电子云密度差异的位置异构体或对映体在界面处的吸附能级差被放大，进而提升热力学分离因子。Sun等^［49］^构建了一种独特的“MOF-金属纳米粒子-COF”三明治结构，其中铂纳米粒子被有效限制在MOF与COF的界面处，这种设计通过双重屏障实现了对催化反应底物尺寸的选择性筛分。近期，Matsumura等^［50］^提出了一种序列可控的MOF-on-MOF生长策略（图4），该研究通过调控外层骨架的生长厚度与配体化学性质，直接干预了界面处的孔隙环境与主客体相互作用势能面。相比于通常仅具有单一孔径与化学环境的纯相MOF（如纯M-BDC或纯M-NDC），这种异质外延生长的复合结构通过在界面处引入位阻识别机制，有助于放大异构体间的扩散速率差异，为位置异构体及聚合物混合物的高效分离提供了更丰富的热力学识别位点^［51］^。此外，针对MOF与COF界面结合力弱的问题，研究者也开发了多种化学连接策略，例如aza-MOF@COF、COF/Mn-MOF杂化材料^［52］^，通过aza-Diels-Alder环加成或配位作用^［53］^将MOF与COF连接。为了更直观地揭示不同构筑策略如何通过调控微观结构来解决色谱分离中的关键问题，本文将各类MOFs基复合材料的结构特点、优势局限及代表性应用实例总结于表1中，旨在从“构筑策略-材料结构-色谱性能”的构效关系维度，为理性设计高性能分离介质提供理论参考。

**图4:**
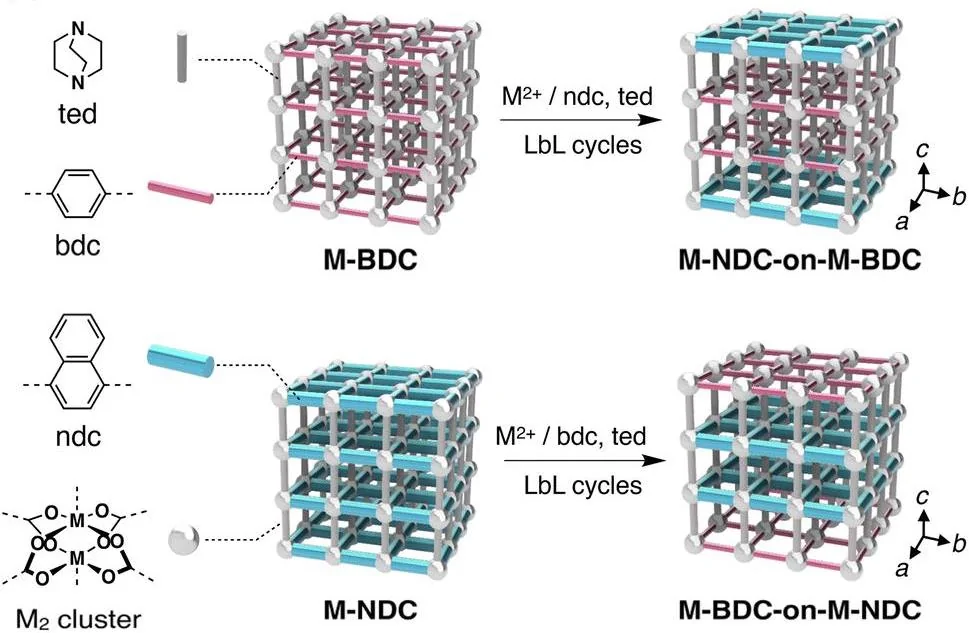
MOF-on-MOF多域晶体的模块化结构^［50］^

**表1:** 不同构筑策略下MOFs基复合材料的性能特点与应用对比

Architecture	Structural features	Advantages & limitations	Typical analytes	Representative composites
Core-shell	functional modification or rigid coating of MOFs via heterogeneous shells （polymers，COFs，SiO_2_，biomacromolecules）	advantages： shell shielding prevents hydrolysis/collapse； introduces meso/macropores to lower diffusion resistance； limitations： multi-step assembly is time-consuming； precise control over shell thickness is challenging	PAHs，flavonoids，enzyme substrates/inhibitors，sulfonamides	SiO_2_@NH_2_-UiO-66@CTF^［54］^，NH_2_-MIL-101（Fe）@COF^［55］^，UiO-66-NH_2_@XOD^［33］^，PCN-224@CTF^［56］^
Host-guest encapsulation	confinement of functional guests （enzymes，ILs，MNPs，macrocycles） within MOF micropores or mesoporous cages	advantages： “confinement effect” prevents guest leaching or aggregation； confers catalytic，chiral，or magnetic functions； limitations： guest size limited by apertures； high loading may block channels and increase backpressure	gas molecules，chiral drugs，alkaloids，positional isomers	CB6@MIL-101^［40］^，Enzyme/MNPs@ZIF-8^［57］^，BHb@PCN-333^［46］^，M-Pc@bio-MOF-1^［45］^
Skeleton hybrid	lattice-level integration of two frameworks via epitaxial growth，interfacial coordination，or cycloaddition	advantages： heterogeneous interfaces induce coupling effects； dual frameworks create complex pore environments to enhance selectivity； limitations： requires high lattice matching； inevitable interfacial defects limit universality	positional isomers，chiral enantiomers，amino acids & antibiotics，complex polymer mixtures	MOF-on-MOF （BDC/NDC）^［50］^，Eu-MOF/HOF^［58］^，NH_2_-MIL-101@CMOM-3S^［59］^，Ti-MOF@Pt@DM-LZU1^［49］^

ILs： ionic liquids； MNPs： metal nanoparticles； PAHs： polycyclic aromatic hydrocarbons.

## 2 MOFs基复合材料在色谱分离中的应用

MOFs基复合材料在色谱领域的研究进展，本质上是将固定相设计从单一的热力学吸附导向转变为兼顾高效传质动力学的综合调控过程。通过构建有序的复合界面，研究者实现了对固定相功能特性的独立调控与集成。在该体系中，MOF组分主要提供高特异性的分子识别位点，而复合基质则负责承担机械支撑、优化流体动力学及调节表面极性。这种多维度的功能重组有效克服了单一材料的性能短板，从而显著拓展了其在复杂体系分析中的分离效率与适用范围。

### 2.1 高效液相色谱中的动力学优化与选择性增强

在HPLC中，固定相需同时满足高压耐受性与快速传质的严苛需求。早期的探索多采用简单的物理涂覆策略，例如将D-His-ZIF-8^［60］^或［Co_2_（D-cam）_2_（TMDPy）］^［61］^简单负载于硅胶表面。这类材料虽然引入了MOF的识别位点，但由于缺乏牢固的界面结合力，不仅柱容量受限，而且难以有效掩蔽硅胶表面残留硅羟基引起的二次相互作用，导致碱性化合物峰形拖尾严重，柱效大幅下降。

为了解决上述问题，近期的复合材料设计更侧重于通过孔道结构的精细调控来突破传质阻力瓶颈。Si等^［62］^构建的d-MOF-818/PAM@silica复合固定相通过缺陷工程在MOF骨架中引入介孔，并利用聚丙烯酰胺网络作为支撑，构建了微孔与介孔贯通的传质通道。这种分级孔道有效缩短了溶质分子的扩散路径，显著降低了范德姆特方程中的传质阻力项，使得该材料在混合模式色谱中克服了纯MOF柱易拖尾的缺点，实现了亲疏水分子的同步高效分离。特别是在亲水相互作用色谱（HILIC）模式下，该复合固定相展现出优于传统商业氨基柱的分离性能，对肌苷的色谱柱效高达8 763.6 plates/m。此外，该材料表现出优异的化学稳定性，在强酸（pH 0.92）及强碱（pH 10.5）等苛刻条件下，经120次连续进样后色谱性能未见明显衰减。

在提升异构体选择性方面，核壳型结构展现了独特的优势。Zhang等^［54］^构建的SiO_2_@NH_2_-UiO-66@CTF杂化材料利用共价三嗪框架（CTF）的刚性大*π*平面包裹MOF核心（图5），实现了内吸附与外筛分相结合的分离机制。外部CTF层对多环芳烃（PAHs）平面结构产生强烈的*π-π*相互作用，而内部MOF层则提供高容量吸附位点。该固定相对萘、芴、菲等结构相似物的分离度显著优于传统C18柱，其理论塔板数高达49 369 plates/m，最高可达59 030 plates/m，而经典C18柱的理论塔板数一般在20 000~40 000 plates/m范围内，充分证实了通过界面互补策略强化材料特异性识别功能的有效性。

**图5:**
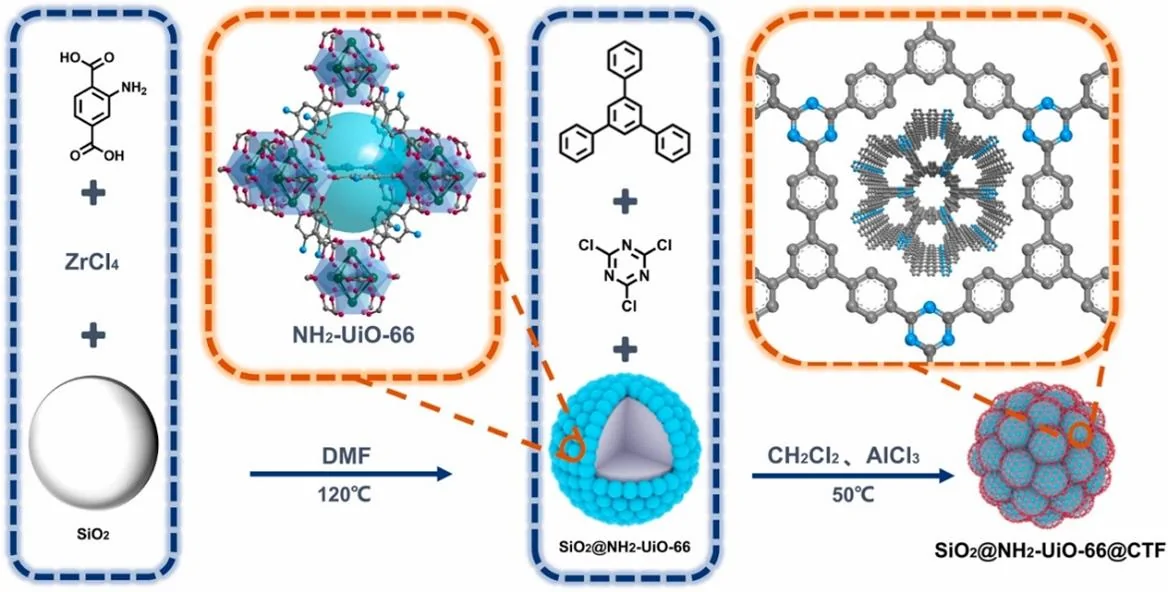
SiO_2_@NH_2_-UiO-66@CTF合成方案^［54］^

此外，针对复杂基质的干扰，将复合材料的高选择性吸附用于在线或离线联用前处理，是提升色谱整体分离效率的另一重要路径^［28，63］^。Ma等^［55］^合成的NH_2_-MIL-101（Fe）@TAPB-FPBA-COF核壳材料，利用外部疏水COF壳层有效排斥果汁基质中的水溶性干扰物，同时利用内部MOF核对黄酮类化合物进行高特异性富集。值得注意的是，该复合吸附剂仅需5 mg即可达到理想回收率，相比传统C18材料（需200 mg）展现出更高的吸附效率。这种兼具抗干扰与强富集能力的机制，结合后续HPLC分析，显著降低了方法的检出限。类似地，Tang等^［64］^利用MOF@COF骨架杂化材料对食品中黄曲霉毒素进行分散固相萃取，结合HPLC-MS/MS检测，充分发挥了复合材料在痕量分析中的富集倍增效应。

### 2.2 毛细管电色谱中的分离选择性与电渗流调节

CEC融合了电泳的高效与色谱的选择性，其核心挑战在于固定相涂层的均匀性及电渗流（EOF）的稳定性。MOFs基复合材料通过引入丰富的表面电荷与作用位点，为调节EOF方向与流速、提升分离选择性提供了新的解决方案^［65-67］^。

在多重相互作用机制的构建上，Ye课题组^［58］^利用化学键合将铕基MOF与氢键有机框架（HOF）复合涂覆于毛细管内壁。该复合涂层集成了MOF的配位位点与HOF的氢键网络，通过双重识别机制实现了对6种磺胺类药物的基线分离。另一种策略是构建骨架杂化复合材料，例如TpBD/UiO-66-NH_2_复合涂层^［68］^利用COF的有序大孔通道促进传质，并结合MOF的氨基位点调节EOF方向，实现了13种氨基酸及复杂抗生素混合物的快速分离。在手性拆分领域，复合界面的微环境调控起到了重要作用。Miao等^［59］^采用MOF-on-MOF外延生长策略制备了NH_2_-MIL-101（Fe）@CMOM-3S，利用复合界面产生的非对称手性微环境，使苯基芳香化合物的对映体分离因子较单一MOF柱获得了显著提升。除了无机骨架间的杂化，构建“MOF-生物分子”生物复合界面是拓展单一MOF功能的另一有效途径。针对纯MOF在手性拆分中常面临的手性配体负载量低及结合不稳定性问题，本课题组Wang等^［69］^近期提出了一种“刚柔耦合”的复合分离界面策略（UiO-66-NH_2_-L-Arg）（图6）。该研究以原位生长的刚性UiO-66-NH_2_为基底，通过共价键合方式在其表面组装了一层柔性L-精氨酸功能网络。这种设计融合了MOF骨架的高比表面积优势与生物分子的特异性识别特性，在显著提升手性配体有效负载密度的同时，利用稳固的共价界面有效抑制了配体流失。实验结果表明，该复合材料基于配体交换机制展现出显著的协同分离效应，成功实现了对多种氨基酸对映体的高效拆分。此外，生物-无机复合体系亦表现不俗，pepsin@MOF-5@poly（GMA-co-EDMA）整体柱^［70］^将胃蛋白酶的生物特异性与MOF的尺寸筛分相结合，在分离羟氯喹等碱性手性药物时，不仅分离度得到改善，且酶的生物活性得到了MOF骨架的有效保护。在生物亲和色谱领域，本课题组Yan等^［33］^基于HP-UiO-66-NH_2_复合涂层毛细管固定化酶微反应器，利用其对传质与反应动力学的有效调节，实现了在线酶分析中底物与产物的快速分离及纳米材料抑制活性的高效筛选。此外，针对样品前处理与CEC的联用，磁性复合材料提供了高效的解决思路。Zhou等^［71］^开发的Fe_3_O_4_@DUT-5磁性微球，集成了磁分离的便捷性与MOF骨架对农药残留的高亲和力，在果蔬样品的磁性固相萃取中展现出优异的回收率，显著提升了后续色谱分析进样溶液的纯度。

**图6:**
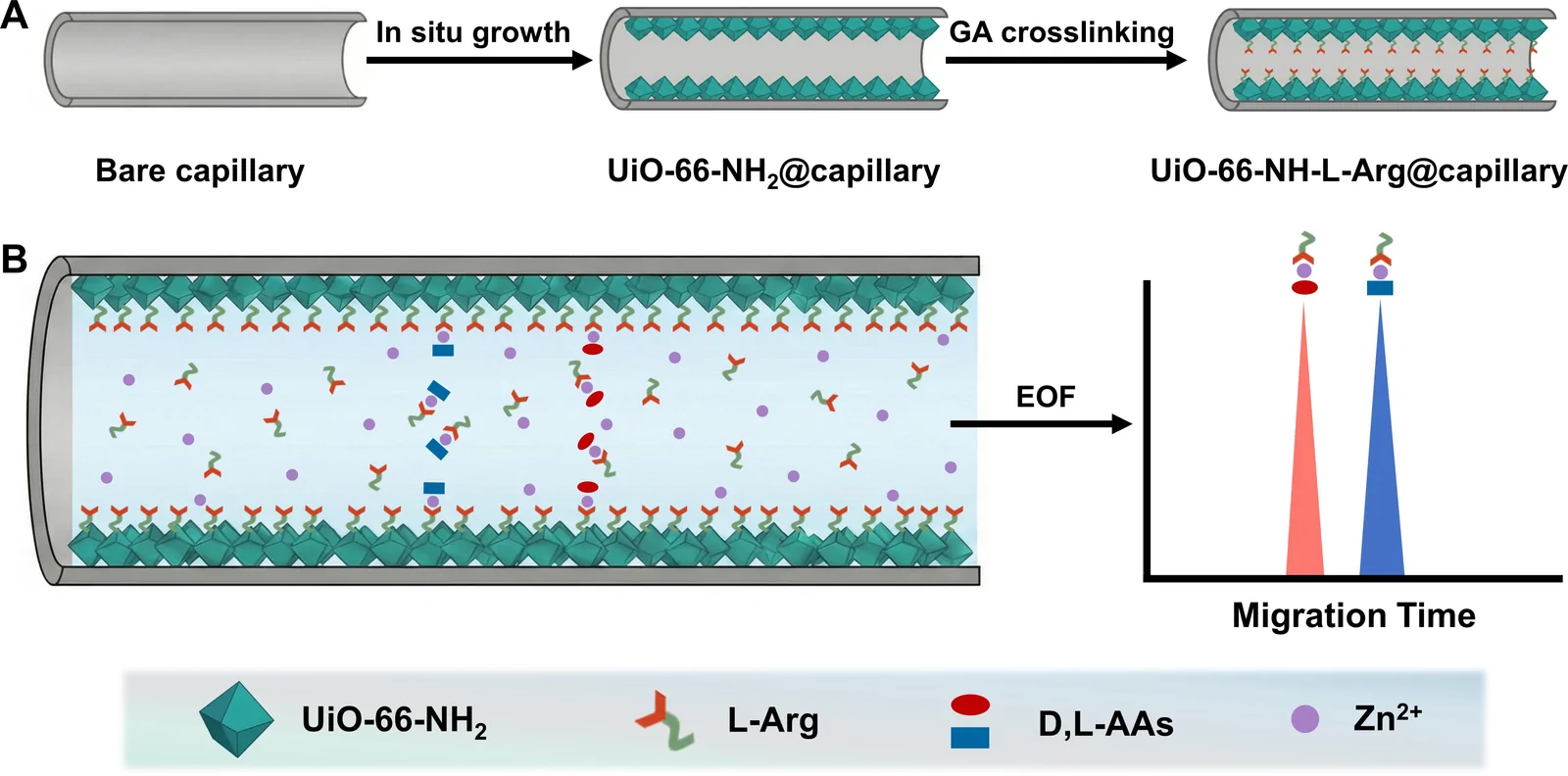
UiO-66-NH-L-Arg毛细管柱合成示意图^［69］^

### 2.3 气相色谱中的耐热性提升与样品富集

GC的高温运行环境是限制配位聚合物长期稳定应用的主要瓶颈之一^［25，72］^，因此该领域的复合材料设计首要解决热稳定性与结构耐受性问题。Liao等^［73］^利用“硫醇-烯”点击化学策略制备的双手性*β*-环糊精基COFs，通过全共价键合网络赋予了材料耐受程序升温的优异热稳定性。热重分析数据显示，该复合骨架在300 ℃之前未表现出明显的质量损失，其热分解温度远高于常规GC分离程序的最高设定温度（通常<280 ℃），成功应用于多种手性药物的高温气相拆分。这一实例不仅证明了多孔框架复合材料完全能够适应GC的严苛条件，更为MOFs基GC分离介质的改性提供了通过复合增强热稳定性的新思路^［74］^。例如，Niu等^［75］^构建的MOF基异质结材料亦表现出类似的热增强效应，其起始分解温度超过400 ℃，有力地证明了复合材料在高温环境下的结构稳定性。

值得注意的是，尽管纯相MOFs作为GC固定相已有相关研究^［76］^，但MOFs基复合材料直接应用于GC固定相的报道仍相对匮乏。这主要归因于GC的高温工况对复合界面的热稳定性提出了苛刻要求：组分间热膨胀系数的失配极易导致涂层在程序升温过程中发生龟裂或剥落。此外，在细径毛细管内实现复合材料的均匀致密成膜也面临较高的技术壁垒。然而，随着界面化学修饰技术的进步，若能有效克服上述热力学匹配与成膜工艺障碍，MOFs基复合材料凭借其多级孔道结构与可调表面性质，有望在复杂难挥发组分的高温气相分离中展现出超越单一材料的潜力。鉴于直接作为固定相的研究尚处于起步阶段，目前将此类复合材料作为气相色谱-质谱（GC-MS）联用的高效前处理介质仍是更为普遍的应用策略。在此场景下，复合材料主要承担高倍率富集的功能。Hu等^［77］^构建了SBA-15@UiO-66-NH_2_核壳复合材料，利用内层介孔SBA-15通道促进大分子快速扩散，同时利用外层UiO-66-NH_2_与磷酸基团间的路易斯酸碱相互作用，实现了对生物样本中痕量糖磷酸盐的高选择性捕获。针对操作便捷性，Al-Maliki等^［78］^设计了Fe_3_O_4_@SiO_2_@Ni，Zn-MOF磁性微球，通过引入磁性内核实现快速磁分离，并利用双金属节点的互补效应及配体间的*π-π*作用，对水体中有机磷农药实现了高达82倍的富集因子，显著提升了GC-FID的检测灵敏度。Kefayati等^［79］^设计的MOF-801@MXene纳米复合涂层则利用MXene的层状结构提供机械支撑并防止MOF颗粒团聚，显著增大了有效吸附面积。在固相微萃取模式下，该涂层对生物样品中的阿片类药物展现出极高的吸附亲和力，结合GC-MS检测实现了较高的灵敏度。此外，针对非极性污染物的分析，Javanmardi等^［80］^通过苯基功能化修饰MOFs，利用“相似相溶”原理增强了材料的疏水性，将其应用于顶空针捕集技术中，有效抵抗了环境水样基质的水分干扰，更实现了对痕量PAHs的超高灵敏度筛查。

## 3 总结与展望

综上所述，MOFs基复合材料的发展代表了色谱固定相设计领域的一个重要演进方向，即从单纯依赖材料筛选逐渐转向对界面性质的结构化调控。本文梳理了该类材料通过引入聚合物网络、无机载体或异质骨架，在改善固定相热力学亲和力与动力学传质性能方面的研究进展。这种基于组分互补的构筑策略，在一定程度上缓解了纯相MOFs材料在色谱应用中吸附容量与扩散速率之间的制约关系。在实际应用层面，MOFs基复合材料通过多级孔道的构筑优化了流体动力学行为，展示了其在复杂异构体及手性分离中的应用潜力；同时，作为样品前处理介质，其可通过选择性富集和基质净化降低复杂样品干扰，并与色谱分析技术相结合，提升痕量目标物检测的灵敏度和可靠性。

尽管MOFs基复合材料在分离科学中表现出良好的应用前景，但要将其从实验室研究转化为稳定可靠的实际分析工具，仍需克服一系列关键技术挑战。为推动该领域的进一步发展，未来的研究工作建议关注以下3个方面。

首先是提升材料在复杂色谱环境下的结构稳定性。目前大部分MOFs材料在酸碱、高水相或缓冲盐体系中的化学稳定性仍有待显著提升，且在超高压流体冲刷下涂层的结合牢度面临考验。未来的设计宜探索更稳固的界面锚定方法，例如利用化学键合替代物理涂覆，或通过引入疏水性保护网络增强骨架抗水解能力，以期改善色谱柱在长周期运行中的耐受性。

其次是改善制备工艺的重现性与便捷性。现有的高性能复合材料常依赖于多步修饰或层层组装工艺，这在一定程度上增加了制备成本，并可能影响批次间的一致性。探索更为简便的可控合成策略或通用的表面修饰技术，实现对涂层厚度及均匀性的有效控制，是MOFs基色谱柱走向实际应用的重要前提。

最后是拓展面向复杂体系的定制化应用。针对生物医药及环境监测等领域日益复杂的分析需求，仅依靠常规的色谱保留机制往往难以满足要求。开发具有环境响应性或特异性识别功能的功能化复合材料将是值得探索的方向。例如，设计能够响应pH或温度变化的动态分离界面，或结合分子印迹技术构建专一性识别位点，有望进一步增强色谱技术在解决实际复杂分析问题中的适用性。随着材料制备技术与色谱理论的不断融合，MOFs基复合材料有望成为提升分离选择性与分析效率的有效解决方案之一。
